# A Polydopamine-Coated Gold Nanoparticles Quenching Quantum Dots-Based Dual-Readout Lateral Flow Immunoassay for Sensitive Detection of Carbendazim in Agriproducts

**DOI:** 10.3390/bios12020083

**Published:** 2022-01-29

**Authors:** Xinxin Mao, Yulong Wang, Lan Jiang, Hanxiaoya Zhang, Yun Zhao, Pengyan Liu, Juanjuan Liu, Bruce D. Hammock, Cunzheng Zhang

**Affiliations:** 1 College of Plant Protection, Nanjing Agricultural University, Nanjing 210095, China; m13675117306@163.com (X.M.); JLan9604@163.com (L.J.); 2020802226@stu.njau.edu.cn (J.L.); 2 Key Lab of Food Quality and Safety of Jiangsu Province-State Key Laboratory Breeding Base, Ministry of Agriculture, Institute of Food Safety and Nutrition, Jiangsu Academy of Agricultural Sciences, Nanjing 210014, China; yllzzn@sina.com (Y.W.); hzha734@aucklanduni.ac.nz (H.Z.); zy943229676@163.com (Y.Z.); 20210047@jaas.ac.cn (P.L.); 3 Department of Entomology and Nematology, UCD Comprehensive Cancer Center, University of California, Davis, CA 95616, USA; bdhammock@ucdavis.edu; 4 State Key Laboratory of Food Science and Technology, Nanchang University, Nanchang 330047, China; 5 School of Biology and Food Engineering, Jiangsu University, Zhenjiang 212000, China

**Keywords:** dual-readout, polydopamine, ZnCdSe/ZnS QDs, carbendazim, lateral flow immunoassay

## Abstract

In this study, a fluorometric and colorimetric dual-readout lateral flow immunoassay (LFIA) using antibody functionalized polydopamine-coated gold nanoparticles (Au@PDAs) as a probe was developed for the detection of carbendazim (CBD). Colloidal gold nanoparticles (AuNPs) were coated with polydopamines (PDA) by the oxidation of dopamine to synthesize Au@PDA nanoparticles. The Au@PDA nanoparticles mediated ZnCdSe/ZnS quantum dots (QDs) fluorescence quenching and recovery, resulting in a reverse fluorescence enhancement detection format of CBD. The CBD detection was obtained by the competition between the CBD and the immobilized antigen for Au@PDAs labelled antibody binding, resulting in a significant fluorescence increase and colorimetry decrease corresponded to the concentration of CBD. Dual readout modes were incorporated into the LFIA using the colorimetry signal under natural light and the fluorescence signal under UV light. The cut-off value in the mode of the colorimetric signal and fluorometric signal for CBD detection was 0.5 μg/mL and 0.0156 μg/mL, respectively. The sensitivity of LFIA of the fluorescence mode was 32 times higher than that of the colorimetry mode. There was negligible cross reactivity obtained by using LFIA for the determination of thiabendazole, benomyl, thiophanate-methyl, and thiophanate-ethyl. Consistent and satisfactory results have been achieved by comparing the results of Au@PDAs-QDs-LFIA and liquid chromatography-tandem mass spectrometry (LC—MS/MS) testing spiked cucumber and strawberry samples, indicating good reliability of the Au@PDAs-QDs-LFIA.

## 1. Introduction

Carbendazim (CBD) is a widely used fungicide. It is very difficult to degrade naturally due to its stable chemical structure, which leads to a large amount of harmful residues in the environment. CBD can cause malformation, embryo toxicity, infertility, developmental toxicity, and germ cell death in mammals; hence, it is recognized as a carcinogen [1]. To ensure food safety, the maximum residue limit of CBD in strawberry and cucumber are set at 0.5 mg/kg and 2 mg/kg in China, respectively [2]. At present, the commonly used techniques for the detection of CBD residues mainly include high performance liquid chromatography (HPLC), liquid chromatography-tandem mass spectrometry (LC—MS/MS), liquid chromatography (LC), mass spectrometry (MS), and gas chromatography (GC) [3,4]. Though these instrumental methods provide high accuracy and sensitivity, they require costly equipment, complex sample preparation, long detection time, and skilled operators, which makes them difficult to be used in situ. There is a demand for the approach to be used in situ easily and sensitively. One of such alternatives is the immunoassay based on the specific binding of antibody and antigen [5,6]. Lateral flow immunoassays (LFIAs), for instance, are known for their ease-of-use, quick results, and low cost, and hence have been widely developed for applications in food safety, environment control, and in vitro diagnostics [7,8,9,10]. However, one of the main issues is their relative low detection sensitivity [11,12,13]. Some common strategies to enhance the sensitivities of LFIA include using more powerful labels, magnifying the labelling signals, etc. [14,15,16,17,18,19]. In fact, the signal output is one of the key factors that influences the functionality and sensitivity of the rapid immunoassays [20,21,22,23].

At present, the dual-mode immunoassay is widely used in LFIA to improve detection sensitivity and accuracy. Zhang et al. developed a visual-afterglow dual-mode immunochromatographic strip for 17β-estradiol detection in milk; the cut-off value in the afterglow mode and visual mode for 17β-estradiol detection was 0.5 ng/mL and 10 ng/mL, respectively. The sensitivity of LFIA of the afterglow mode was 20 times higher than that of the visual mode [24]. You et al. developed a colorimetric and fluorescent dual-mode immunochromatographic strip for PSA in whole blood, and the cut-off value of this immunoassay was 1.07 pg/mL, which is at least two orders of magnitude lower than the conventional fluorescence immunoassay [25]. Noble metal nanoparticles [26,27,28], quantum dots (QDs) [29,30], and up-conversion nanoparticles [31,32], etc., are often used as sensitive signal probes. QDs, also known as semiconductor nanocrystals, can provide high fluorescence quantum yields. Their characteristics of wide excitation spectrum, narrow emission spectrum, good biocompatibility, and long florescence time make them ideal analytical probe tracers [33,34]. Polydopamine (PDA), the polymer of dopamine (DA), as a new biomimetic adhesive, is gaining attention in various areas thanks to its excellent chemical reactivity, biocompatibility, quenching effect, and adhesion ability [35,36,37]. PDA is able to attach to the surface of nanoparticles through electrostatic effects or covalent bonds, which results in higher ultraviolet-visible spectroscopy (UV-vis) absorption than the bare nanoparticles without PDA attachment [38,39,40,41]. In this study, gold nanoparticles (AuNPs) were coated with PDA, and the resulted label (Au@PDAs) was expected to provide higher color intensity, colloidal stability, and antibody binding efficacy, which enhances the overall sensitivity of the assay.

In this study, we used QDs as the fluorescent donors and Au@PDAs as the fluorescent acceptors to achieve fluorescence quenching. The quenching of fluorescence is achieved by the fluorescence resonance energy transfer (FRET) between the fluorescence donor and acceptor [42]. During the detection process, Au@PDAs-mAb deposits on the T line due to the combination of antibody and antigen and quenches the fluorescence of ZnCdSe/ZnS QDs when Au@PDAs and the QDs on the T line reach an appropriate distance [43,44]. When the target compound is present during the assay, due to the competition between target and CBD-OVA, the amount of Au@PDAs-mAb captured by CBD-OVA on the T line is significantly reduced, the fluorescence of QDs is recovered, and the fluorescent intensity is proportional to the concentration of the target. Based on the deep original color of Au@PDAs itself and the fluorescence signal of quantum dots, dual readout modes were incorporated into the LFIA using the colorimetry signal under natural light and the fluorescence signal under UV light. The double-readout strategy was adopted to improve the accuracy and sensitivity of the detection. Based on such mechanism, a highly sensitive LFIA could be developed. We applied the strategy to the detection of CBD in cucumber and strawberry.

## 2. Material and Methods

### 2.1. Reagents and Instrumentals

Goat anti-mouse immunoglobulin G (IgG) was from Boster Biological Technology Wuhan Branch (Wuhan, China). Carbendazim (CBD), thiabendazole, benomyl, thiophanate-methyl, and thiophanate-ethyl pesticide standards were obtained from National Standards (Tianjin, China). ZnCdSe/ZnS quantum dots (ZnCdSe/ZnS QDs) were purchased from Wuhan Jiayuan Quantum Dot Technology Development Co., Ltd. (Wuhan, China). Trisodium citrate, bovine serum albumin (BSA), gold chloride trihydrate (HAuCl_4_·3H_2_O), and dopamine hydrochloride (DA·HCl) were obtained from Sigma-Aldrich (St. Louis, MO, USA).

A XYZ3050 dispensing platform was from Bio-Dot (Irvine, CA, USA). A ZQ2000 guillotine cutter was from Jinbiao Biotechnology (Shanghai, China). Hi-Flow Plus HFC13502 membrane (NC membrane) was obtained from Millipore Corp. (Bedford, MA, USA). Glass fiber conjugate pads were obtained from Millipore Corp. (Bedford, MA, USA). Semirigid polyvinyl chloride (PVC) sheets were obtained from Jiening Biotech (Shanghai, China). Absorbent pads were obtained from Jiening Biotech (Shanghai, China).

### 2.2. Preparation of CBD-BSA

In this study, we used CBD hapten, which was synthesized in our laboratory. The conjugation of CBD hapten and BSA carrier protein was achieved via EDC-NHS coupling. Briefly, a mixture of 9 mg CBD hapten, 13.8 mg EDC, and 6 mg NHS was dissolved in 0.5 mL DMF (solution A). The mixture was stirred at room temperature overnight. Then, solution A was added dropwise to BSA (60 mg) in 5 mL PBS (0.01 M, pH 7.4). The mixture was stirred at room temperature for 8 h and then dialyzed in 1 L PBS (0.01 M, pH 7.4) for 3 days and stored at –20 °C. The UV-vis spectra among artificial antigens, BSA, and the hapten were compared. Matrix-assisted laser desorption time-of-flight (MALDI-TOF) was used to measure the hapten-to-protein molar ratio, and the coupling ratio was evaluated by the following equation:

$$Coupling\;ratio=\frac{MW_{conjugage}-{MW}_{protein}}{MW_{hapten}}$$

### 2.3. Production of Anti-CBD Monoclonal Antibody

The immunization strategy was detailed in Table S1. Ten Balb/c female mice (6–8 weeks old) were immunized with 50 μg of immunogens (CBD-BSA) in Freund’s complete adjuvant via intraperitoneal injection. In the next four sequential immunizations, the immunogen emulsified with Freund’s incomplete adjuvant was given to each mouse in the same way; the time interval for each immunization was two weeks. One week after the third, fourth, and fifth immunization, the serum was collected from each mouse and was monitored by indirect competitive ELISA (icELISA). The mouse with the most sensitive antibody was selected for the cell fusion. The SP2/0 myeloma cells were fused with mouse spleen cells using 50% PEG and were cultured in HAT medium. The culture supernatants were detected by icELISA. The hybridomas that produced anti-carbendazim monoclonal antibody (anti-CBD mAb) were subcloned using the limiting dilution method. After three rounds of subcloning, the subcloned hybridomas were collected and used for the production of ascites. The ascites were purified by protein G and used for the development of icELISA.

### 2.4. Preparation of Colloidal Gold

The monodispersed AuNPs with average particle size of 20–25 nm were prepared according to a previous report [45]. Briefly, 0.5 mL 2% (*w*/*v*) HAuCl_4_ solution was added to 99.5 mL ultrapure water and was heated to boiling with a magnetic stirrer. Then, 2.4 mL of 1% (*w*/*v*) sodium citrate was quickly added and was boiled for another 5 min until the solution appear to gradually change color, turning from gray to orange or red. After cooling to room temperature, the AuNP solution was adjusted by topping it up with ultrapure water to volume of 100 mL. The AuNP solution was then characterized by UV–vis spectra at 400 nm and 600 nm. The dispersibility and diameter of AuNPs were evaluated by transmission electron microscopy (TEM). The solution was stored at 4 °C.

### 2.5. Preparation of Au@PDA Nanoparticles

Au@PDAs were synthesized according to a previous report [46]. Briefly, 5 mL AuNP solution was centrifuged at 10,000 rpm for 30 min. The supernatant was discarded, and the precipitation was resuspended with 5 mL Tris-HCl (0.01 M, pH 8.5). The solution was divided equally into 5 portions (1 mL each), and each portion was added with 4 μL 3% H_2_O_2_ and 0, 50, 100, 150, 200 μL DA·HCl (5 mg/mL), respectively. The mixtures were stirred to react in the dark for 1 h at room temperature. After the reaction, each portion was centrifuged for 30 min at 10,000 rpm and was resuspended with 1 mL ultrapure water. As a result, solutions of Au@PDAs with different dopamine layer thickness were obtained.

### 2.6. Conjugation of QDs-OVA

In a brown flask, 100 μL surface carboxylated ZnCdSe/ZnS QDs (diluted with boric acid buffer, 800 nM, pH = 6) was mixed with 2.4 μL EDC (10 mg/mL). The mixture was stirred at room temperature overnight in the dark. Then, 10 μL OVA (30 mg/mL) was added to the mixture, which was stirred at room temperature overnight in the dark. The QDs-OVA (800 nM) conjugates were then obtained.

### 2.7. Preparation of Au@PDAs-mAb Probe

The Au@PDAs-mAb probe was prepared as described in a previous report [47]. First, the optimal pH of the Au@PDA solution and the amount of mAbs for conjugation were determined. Briefly, different amounts of K_2_CO_3_ (0.1 M) were added to several vials of 1 mL Au@PDA solutions. The pH of each solution was 5, 6, 7, 8, and 9, respectively. Then, 10 μL anti-CBD mAb was added to each solution (1.01 mg/mL), which was stirred at room temperature for 1 h. Then, 10 μL 10% PEG-20000 (*m*/*v*) was then added to each solution, which was stirred gently at room temperature for 30 min, followed by adding 10 μL 10% BSA (*m*/*v*) and stirring gently at room temperature for 30 min. Afterwards, the solutions were centrifuged at 10,000 rpm at 4 °C for 30 min. The precipitates were resuspended with 100 μL ultrapure water to obtain the probes and stored at 4 °C for further use. The optimal pH was selected based on the performance of the test at each pH level. Au@PDAs were then adjusted to the optimum pH. Then, 5, 10, 15, and 20 μL anti-CBD mAb (1.01 mg/mL) was added to 1 mL Au@PDAs, respectively. The same procedure was repeated with different antibody labeling amounts to determine the optimal antibody labeling amount. Finally, the optimized probes were produced by adjusting the pH and antibody labeling amount to the optimal values in the procedure.

### 2.8. Fabrication of the Au@PDAs-QDs-LFIA Test Strips

The test strip consisted of a nitrocellulose (NC) membrane, sample pad, absorbent pad, and conjugate pad. On the NC membrane, the capture reagent (CBD-OVA and QDs-OVA) were sprayed as the test line (T line), and goat anti-mouse IgG was sprayed as the control line (C line). After spraying, the NC membrane was dried at 37 °C for 30 min. The sample and conjugate pads were treated with the blocking buffer and dried at 37 °C for 24 h. The sample pad, conjugate pad, NC membrane, and absorbent pad were laminated and pasted on a PVC sheet. Finally, the assembled scaleboard was cut into strips (60 mm × 3.5 mm) by the ZQ2000 guillotine cutter and dried at 37 °C for 30 min.

Then, 0.75 μL of probe and 100 μL of sample solution were premixed in the well for 2 min, and then the strip was inserted. After 15 min, the detection result was determined by naked eye.

### 2.9. Elimination of Sample Matrix Interference

The cucumber and strawberry without CBD were ground into paste in a mortar, and 5 g of each sample was weighed and put into 50 mL centrifuge tubes, and then 10 mL acetonitrile and 2 g NaCl were added to each centrifuge tube, which were shaken for 30 min on a shaking table. Afterwards, the sample were centrifuged at 5000 rpm at 4 °C for 5 min. Then, 5 mL of the upper extract was absorbed into a 10 mL centrifuge tube and dried with nitrogen. After drying with nitrogen, 2 mL of sample diluent was added to replace the organic extraction solvent; the complex solution was diluted 2-, 5-, 10-, and 20-fold with the sample diluent. The amounts of 0, 250, and 500 ng/mL CBD were then added to these sample dilutions. A control group was prepared by adding 0, 250, and 500 ng/mL CBD to the working buffer (5% methanol PBS). According to the test strip result, the best sample dilution times were selected to eliminate the interference of the sample matrix.

### 2.10. Method Validation

Pre-treatment of samples was conducted for LC—MS/MS. Strawberry and cucumber were ground into paste in a mortar, 5 g of each sample were weighed and put it into 50 mL centrifugal tubes, and CBD with certain concentrations was added in the centrifuge tubes, which were 0, 0.25, 0.5, 1 and 0, 1, 2, 4 mg/kg, respectively. The samples were shaken and mixed on the shaking table for 10 min. Then, 10 mL acetonitrile and 2 g NaCl were added to each centrifuge tube, which were shaken and mixed on the shaking table for 30 min again. Afterwards, the solutions were centrifuged at 5000 rpm at 4 °C for 5 min, and the upper extract solutions of cucumber and strawberry were passed through a 0.22 μm filter membrane for LC—MS/MS detection.

Pre-treatment of samples for the test strip was conducted. The treatment process was the same as above. First, 5 mL of the upper extract was absorbed into a 10 mL centrifuge tube; after nitrogen drying, 2 mL sample diluent was added to replace the organic extraction solvent, and the best dilution time to eliminate matrix interference was selected for the strip test. The test results of the strip test were compared with the test results of LC—MS/MS, which proved that the test strip test results were reliable.

### 2.11. Detection of Real Samples

To evaluate the reliability of Au@PDAs-QDs-LFIA, 20 real strawberry and cucumber samples from a farmer’s market were detected by Au@PDAs-QDs-LFIA and LC—MS/MS. The sample treatment process was the same as method validation.

## 3. Results and Discussion

### 3.1. Production of Monoclonal Antibody and the Preparation of Detection Probes

For the purpose of obtaining specific antibody to target CBD, hapten should preserve the parent structure of CBD, while a space arm with an active group is essential to maximumly exposure the parent structure. Previous studies showed that the molecular structure derived from aminobenzimidazole with a six-carbon-chain link arm was a good hapten to induce a specific antibody against CBD [48]. Moreover, the classical immunological study reveals that molecules smaller than 5000 Da are usually conjugated as haptens to large molecular weight carriers (usually proteins) to induce antibody formations [49]. BSA is an ideal carrier because of its relatively low price and abundant lysine residues [50]. Thus, we coupled the hapten to BSA as an immunogen (CBD-BSA). The maximum absorption peaks of the hapten, BSA, and artificial antigen were at 286 nm, 277 nm, and 282 nm, respectively (Figure S1A). The coupling ratio of CBD hapten to BSA was calculated at 27:1 by using MALDI-TOF (Figure S1B). These results indicated that the immunogen was successfully synthesized.

**Figure 1:**
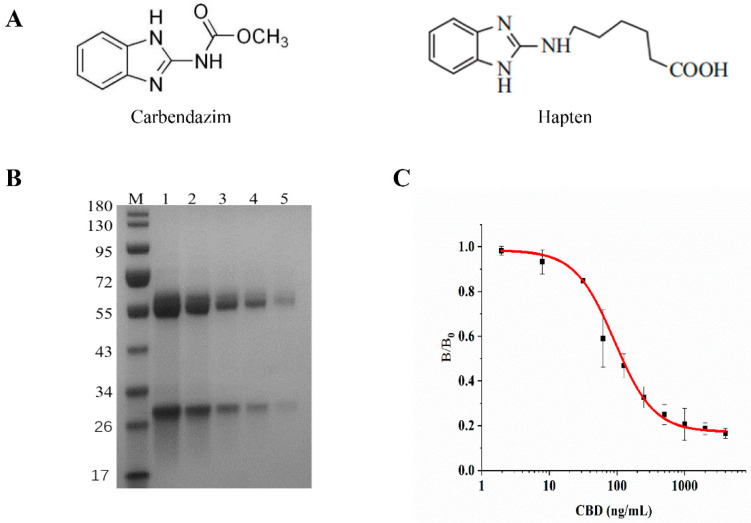
(**A**) CBD and CBD hapten. (**B**) Gel electrophoresis of anti-CBD mAb (lane M: protein marker; lane 1–5: different concentrations of anti-CBD mAb). (**C**) Inhibition curve of anti-CBD mAb to CBD.

Balb/c female mice were immunized with the CBD-BSA immunogen for mAb production. Mouse no. 3 was sacrificed for cell fusion (Figure S2). The cell line producing the specific antibody against CBD was obtained and was named 5E. The purity of the anti-CBD mAb was verified via SDS-PAGE; only two bands were shown on the lane, which represented the heavy chain (60 kD) and light chain (30 kD) that were broken down from the anti-CBD mAb during the heat treatment. There were no miscellaneous protein bands visible (Figure 1B). The sensitivity of antibody was determined by icELISA. The dose–response standard curve showed that at the optimal working concentrations, the linear range was 21.45–856.05 ng/mL (IC_20_–IC_80_), and IC_50_ was 119.28 ng/mL for CBD (Figure 1C).

**Figure 2:**
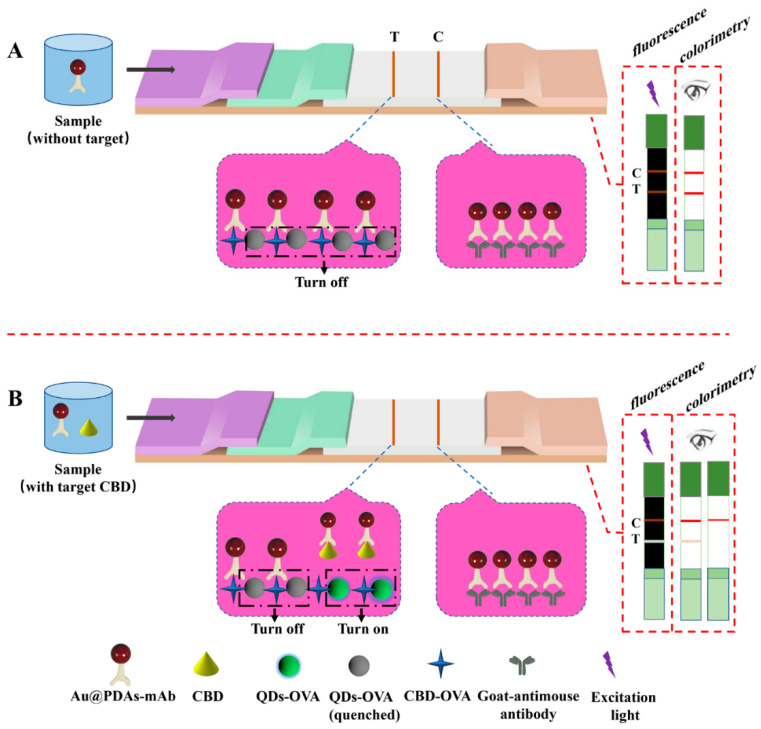
Principle of Au@PDAs-QDs-LFIA: (**A**) detection mode without target CBD; (**B**) detection mode with target CBD.

The optical performance and stability of detection probes (Au@PDAs-mAb and QDs-OVA) are important to the LFIA system. In the LFIA system, Au@PDAs-mAb can be captured by CBD-OVA at T line; thus, captured Au@PDAs-mAb will not only show an obvious red color, but will also quench the fluorescence of ZnCdSe/ZnS QDs due to a fluorescent resonance energy transfer (FRET) from ZnCdSe/ZnS QDs to the captured Au@PDAs (Figure 2). An overlap between the emission spectrum of the donor and the absorption spectrum of the acceptor is the prerequisite for FRET-based quenching [51]. The test strip interpretation standard is shown in Figure S3. The UV-vis absorption peak of the Au@PDAs-mAb developed in this study is 527 nm, while the emission peaks of the photoluminescence spectra of ZnCdSe/ZnS QDs and QDs-OVA are 522 nm (Figure S4), indicating that there is a significant overlap between the two spectra, and effective FRET could be achieved.

**Figure 3:**
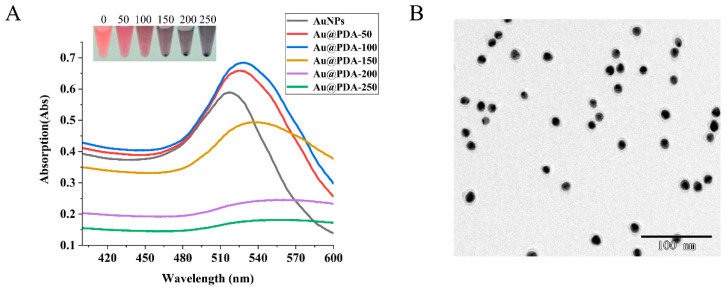
(**A**) UV–vis absorption spectra of AuNPs, Au@PDA-50, Au@PDA-100, Au@PDA-150, Au@PDA-200, and Au@PDA-250. (**B**) TEM image of Au@PDA-100.

PDA attached to the surface of nanoparticles resulted in higher UV–vis absorption than the bare nanoparticles, as a darker color was displayed in the colorimetry mode of the LFIA. However, a PDA layer of appropriate thickness could increase the absorbance of Au@PDAs, while a thick PDA layer would cause the aggregation of Au@PDAs, resulting in a wider absorption peak and lower absorbance [52]. Thus, we compared various layers of Au@PDAs in terms from stability and absorption spectrum. The UV–vis results (Figure 3A) show that the maximum absorption peaks of AuNPs, Au@PDA-50, Au@PDA-100, Au@PDA-150, Au@PDA-200, and Au@PDA-250 were 518, 525, 529, 537, 542, and 547 nm, respectively. As the volume of DA·HCl increased from 50 μL to 100 μL, the maximum absorbance of Au@PDAs increased from 0.6587 to 0.6846. As the volume of DA·HCl continued to increase, the maximum absorbance of Au@PDAs decreased to 0.1807. Therefore, Au@PDA-100 was chosen as the antibody marker. The TEM image (Figure 3B) shows that Au@PDA-100 had good dispersibility and uniformity, with an average particle size of 20 nm. In addition, Figure S5 reveals that the Au@PDAs-mAb and QDs-OVA solutions with different storage times had good colloidal stability and optical stability.

### 3.2. Assay Optimization

We performed a series of optimization experiments, including the pH value of the Au@PDA solutions, concentrations of CBD-OVA and QDs-OVA, and the volume of Au@PDAs-mAb, to attempt to achieve better sensitivity and to characterize the LFIA for further applications. An appropriate labeling strategy with high binding efficiency of mAb was evaluated first by optimizing the pH value and the amount of mAb loaded on Au@PDAs. As it turned out, the color of the T line was the clearest when the pH was 6, slightly more intense than it when the pH was 5, 6, 7, 8, and 9 (Figure S6A). Hence, 6 was the optimized pH for labeling the mAb. The amount of mAb loaded on Au@PDAs directly affected the dispersion and stability of the probe, thereby affecting the sensitivity of detection. The optimal antibody labeling amount was 10 μL, that is, the optimal antibody labeling concentration was 10.1 μg/mL (Figure S6B).

In competitive LFIA, a competitor antigen (CBD-OVA) competed with the analyte for the limited binding sites presented on the Au@PDAs-mAb, and the concentration of CBD-OVA and the volume of Au@PDAs-mAb had significant effects on assay sensitivity. The concentrations of the coating antigen (CBD-OVA) on the T line were set to 0.5, 0.6, 0.8, and 1.0 mg/mL. It turned out that 0.8 mg/mL was the optimized concentration of the coating antigen, as the color intensity of the T line was the closest to that of the C line (Figure S7A). The concentrations of QDs-OVA sprayed on the T line were set to 200, 400, and 600 nM. The concentration was the most suitable if the T line was moderately fluorescent when there was no probe and appeared red naturally, but the fluorescence was quenched when the probes were loaded. As shown in Figure S7B, 400 nM was the concentration that fulfilled such condition. The fluorescence was moderate and completely quenched after adding 0.75 μL of probes. In comparison, the fluorescence intensity was too low at 200 nM and too high at 600 nM. Therefore, the optimized conditions were 0.8 mg/mL CBD-OVA, 400 nM QDs-OVA, and 0.75 μL Au@PDAs-mAb probes.

**Figure 4:**
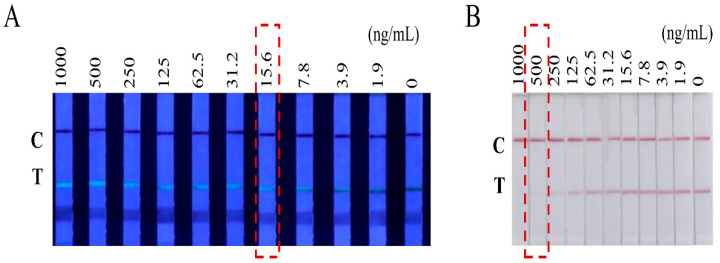
Cut-off values of the CBD test strip. (**A**) Test strips under ultraviolet light with different concentrations of CBD. (**B**) Test strips under natural light with different concentrations of CBD.

Under the optimized conditions, CBD was diluted with 5% methanol PBS to several concentrations, which were 1000, 500, 250, 125, 62.5, 31.2, 15.6, 7.8, 3.9, and 0 ng/mL. The test results showed that the cut-off value (the minimum CBD concentration that makes T line invisible) of CBD at the fluorescence mode under ultraviolet light was 15.6 ng/mL (Figure 4A). The detection cut-off value of CBD under natural light was 500 ng/mL (Figure 4B). In addition, we used ImageJ software to translate the band intensity to a quantitative value. Standard curves were built with good linear relation in both modes, and the LOD and linear range were, respectively, 1.36 ng/mL and 3.56–246.67 ng/mL for fluorescence mode, while the LOD and linear range were, respectively, 3.21 ng/mL and 7.21–945.55 ng/mL for colorimetry mode (the quantified graphs are not shown). The sensitivity of the assay was comparable to the sensitivity of reported immunodetection methods (Table S2).

**Figure 5:**
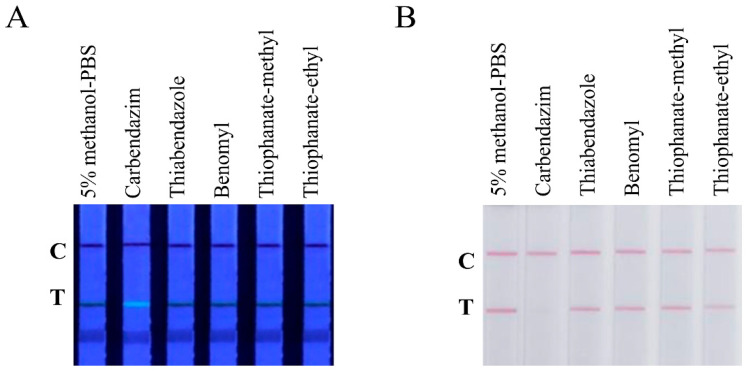
Specificity of the test strip. (**A**) Strips added with CBD, thiabendazole, benomyl, thiophanate-methyl, and thiophanate-ethyl under ultraviolet light. (**B**) Strips added with CBD, thiabendazole, benomyl, thiophanate-methyl, and thiophanate-ethyl under natural light.

CBD, thiabendazole, benomyl, thiophanate-methyl, and thiophanate-ethyl were all diluted to 1 μg/mL with 5% methanol PBS and were added to Au@PDAs-QDs-LFIA test strips. Only the test strips added with CBD gave positive results, and the results of thiabendazole, benomyl, thiophanate-methyl, and thiophanate-ethyl were all negative (Figure 5). Therefore, the Au@PDAs-QDs-LFIA test strip was very specific to CBD and would not be affected by other fungicides in practical detection scenarios.

### 3.3. Sample Analysis

Different diluents were tested to optimize the sample conditions, including dH_2_O, Tris-HCl, PBS, and PBST. Among the 4 diluents, PBS appeared to be the most suitable, as the colors on C line and T line were minimally affected (Figure S8A). Organic solvents are often used to improve analyte solubility or as a part of the sample preparation procedure. PBS with different amounts of methanol (0%, 5%, 10%, and 20%) was optimized. These methanol PBS diluents were used to prepare strawberry and cucumber samples spiked with CBD (0, 0.5, and 1.0 μg/mL). As shown in Figure S8B, when methanol was at 5%, the colors on the C line and T line were minimally affected, and no false positive or negative appeared. The pH of the diluent was then adjusted (6, 7, 8, 9, and 10). Then 5% methanol PBS with different pH was used to prepared strawberry and cucumber samples spiked with CBD (0, 0.5, and 1.0 μg/mL). As shown in Figure S8C, when the pH was 7, the colors on the C line and T line were minimally affected, and no false positive or negative appeared.

The matrix of real samples is actually different from the standard solutions. It could interfere with the antibody–antigen recognition, thus generating an unmatched signal response of analytes. Diluting the real sample with the standard diluent is an efficient way to decrease the matrix effect. After the cucumber and strawberry samples were dissolved in 5% methanol PBS with the dilution factors of 5, 10, and 20 times, they were spiked with CBD. The diluted samples were then run on the test strips. The results showed that the sample matrix interference could be eliminated when the dilution factor was greater than or equal to 10 times (Table 1).

The results of LC—MS/MS and the Au@PDAs-QDs-LFIA test strip were compared. The average recovery ratio detected by LC—MS/MS was 74.5–102.6%, and the relative standard deviation was less than 7.2%. The results of the test strip were very similar to those of LC—MS/MS, which proved that the test strip test results were reliable (Table 2).

Table 3 shows the detection results of 20 real strawberry and cucumber samples purchased from a market using Au@PDAs-QDs-LFIA and LC—MS/MS. Among these samples, three samples (Strawberry: Nos. 7, 9; Cucumber: No. 2) detected by Au@PDAs-QDs-LFIA were positive using the fluorescence mode (ultraviolet light), but negative using the colorimetry mode (natural light), respectively, whereas those detected by LC—MS/MS were 23 ± 0.015, 48 ± 0.076, and 16 ± 0.036 ng/mL, respectively. The detection results of Au@PDAs-QDs-LFIA were in agreement with those of LC—MS/MS.

## 4. Conclusions

In this study, a highly sensitive Au@PDAs-QDs-LFIA was developed based on Au@PDAs. The LFIA was able to rapidly and accurately detect CBD residues in cucumber and strawberry samples, the results of which were verified with LC—MS/MS to prove the reliability. The detection results of real strawberry and cucumber samples by Au@PDAs-QDs-LFIA were in agreement with LC—MS/MS. The CBD detection cut-off value of the LFIA was 0.0156 μg/mL using the fluorescence mode (ultraviolet light) and 0.5 μg/mL using the colorimetry mode (natural light). The sensitivity using the fluorescence mode was increased by 32 times compared with it using the colorimetry mode. At the same time, this LFIA showed no cross-reactivity with thiabendazole, benomyl, thiophanate-methyl, and thiophanate-ethyl. Therefore, the Au@PDAs-QDs-LFIA developed in this study provides high sensitivity and specificity, which has potential to be applied in various practical detection scenarios.

## Figures and Tables

**Table 1:** Matrix interference on test strip.

Sample	Concentration of CBD (ng/mL)	5% Methanol-PBS		Dilution							
		Colorimetry Signal	Fluorescence Signal	Colorimetry Signal				Fluorescence Signal			
				2×	5×	10×	20×	2×	5×	10×	20×
Strawberry	0	–	–	–	–	–	–	–	–	–	–
	250	±	+	–	±	±	±	–	±	+	+
	500	+	+	–	±	+	+	–	±	+	+
Cucumber	0	–	–	–	–	–	–	–	–	–	–
	250	±	+	–	±	±	±	–	±	+	+
	500	+	+	–	±	+	+	–	±	+	+

Colorimetry signal (T line): –, an obvious red band was observed; ±, the red band was light; +, no red band was observed. Fluorescence signal (T line): –, no fluorescence band was observed; ±, the fluorescence band was light; +, an obvious fluorescence band was observed.

**Table 2:** Test results of strip test and average recoveries of LC—MS/MS for CBD in spiked samples.

Sample	Concentration of CBD (mg/kg)	Au@PDAs-QDs-LFIA		LC—MS/MS		
		Colorimetry Signal	Fluorescence Signal	Found (mg/kg)	Recovery (%)	CV (%)
Strawberry	0.25	–	+	0.247	98.6	2.9
	0.5	±	+	0.450	90.1	6.2
	1	±	+	0.816	81.6	2.8
Cucumber	1	±	+	0.872	87.2	3.1
	2	±	+	1.58	79.0	5.2
	4	+	+	3.708	92.7	4.8

Colorimetry signal (T line): –, an obvious red band was observed; ±, the red band was light; +, no red band was observed. Fluorescence signal (T line): +, an obvious fluorescence band was observed.

**Table 3:** Detection results of real strawberry and cucumber samples using this method and LC—MS/MS.

Sample	Sample Number	Au@PDAs-QDs-LFIA		LC—MS/MS
		Colorimetry Signal	Fluorescence Signal	Found (ng/mL)
Strawberry	1	-	-	ND
	2	-	-	ND
	3	-	-	ND
	4	-	-	ND
	5	-	-	ND
	6	-	-	ND
	7	-	+	23 ± 0.015
	8	-	-	ND
	9	-	+	48 ± 0.076
	10	-	-	ND
Cucumber	1	-	-	ND
	2	-	+	16 ± 0.036
	3	-	-	ND
	4	-	-	ND
	5	-	-	ND
	6	-	-	ND
	7	-	-	ND
	8	-	-	ND
	9	-	-	ND
	10	-	-	ND

ND: not detected.

## Data Availability

This study did not report any data.

## Supplementary Materials

The following supporting information can be downloaded at: www.mdpi.com/xxx/s. 1Immunization and blood collection program of mouse (Table S1), comparison of other testing methods for the detection of CBD reported in literatures (Table S2), the characterization of immunogen CBD-BSA (Figure S1), effect of polyclonal antibody serum in immunized mouse (Figure S2), schematic diagram of Au@PDAs-QDs-LFIA test strip for result judgment (Figure S3), photoluminescence spectra of ZnCdSe/ZnS QDs and QDs–OVA (Figure S4), UV-vis absorption spectrum of Au@PDAs-mAb and photoluminescence spectrum of QDs-OVA (Figure S5), optimal pH value and amount of mAb for detection probe (Figure S6), optimization of test strip spraying conditions (Figure S7), optimization of test conditions for strip samples (Figure S8). References [53,54,55,56] are cited in the supplementary materials.
