# Supplementary material for: A Polydopamine-Coated Gold Nanoparticles Quenching Quantum Dots-Based Dual-Readout Lateral Flow Immunoassay for Sensitive Detection of Carbendazim in Agriproducts
Source: Biosensors (Basel). 2022 Jan 29;12(2):83. doi: 10.3390/bios12020083 (PMC8869244; doi:10.3390/bios12020083)
Supplement: Supplementary file 1 [file biosensors-12-00083-s001.zip › biosensors-1557032-supplementary.pdf]

biosensors

Supporting Information

A Polydopamine-Coated Gold Nanoparticles Quenching Quantum Dots-Based Dual-Readout Lateral Flow Immunoassay for Sensitive Detection of Carbendazim in Agriproducts

Xinxin Mao [1,2,†], Yulong Wang [2,†], Lan Jiang [1], Hanxiaoya Zhang [2], Yun Zhao [2], Pengyan Liu [2], Juanjuan Liu [1], Bruce D. Hammock [3] and Cunzheng Zhang [1,2,4,5,*]

Immunization Strategy

Balb/c female mice of 6–8 weeks of age were immunized with the synthesized CBD-BSA immunogen. The immunization strategy was detailed in Table S1.

Table S1. Immunization and blood collection program of mouse.

Time	Aadjuvant	CBD-OVA dose (μg/mouse)	Site
Blood collection (day 1)	-	-	Tail
First immunization (day 8)	Freund's complete adjuvant	50 μg	Intraperitoneal
Secondary immunization (day 29)	Freund's incomplete adjuvant	50 μg	Intraperitoneal
Third immunization (day 43)	Freund's incomplete adjuvant	50 μg	Intraperitoneal
Blood collection (day 50)	-	-	Tail
Fourth immunization (day 57)	Freund's incomplete adjuvant	50 μg	Intraperitoneal
Blood collection (day 64)	-	-	Tail
Fifth immunization (day 71)	Freund's incomplete adjuvant	50 μg	Intraperitoneal
Blood collection (day 78)	-	-	Tail
Booster immunization (day 85)	Saline	100 μg	Intraperitoneal
Cell fusion (day 88)	-	-	Remove the spleen

Comparison of Different Immunoassays for CBD

Compared with other quantitative detection methods reported in literature, the immunochromatographic strip prepared in this study has obvious advantages, based on the deep original color of Au@PDAs itself and the fluorescence signal of quantum dots, dual readout modes were incorporated into the LFIA. The double-readout strategy is adopted to improve the accuracy and sensitivity of the detection. As shown in Table S2.

Table S2. Comparison of other testing methods for the detection of CBD reported in literatures.

Method	LOD (mg/kg)	Time (min)	Reference
ELISA	0.815	40-60	[53]
GICA	0.300	15	[54]
Electrochemical	5.736×10^{-4}	40	[55]
Spectrophotometry	0.026	30-60	[56]
Au@PDAs-QDs-LFIA	0.125	15	This work

Characterization of the CBD-BSA

The maximum absorption peak of the artificial antigens deviated from BSA and hapten (Figure S1A). The hapten-to-protein molar ratio was identified by using MALDI-TOF (Figure S1B).

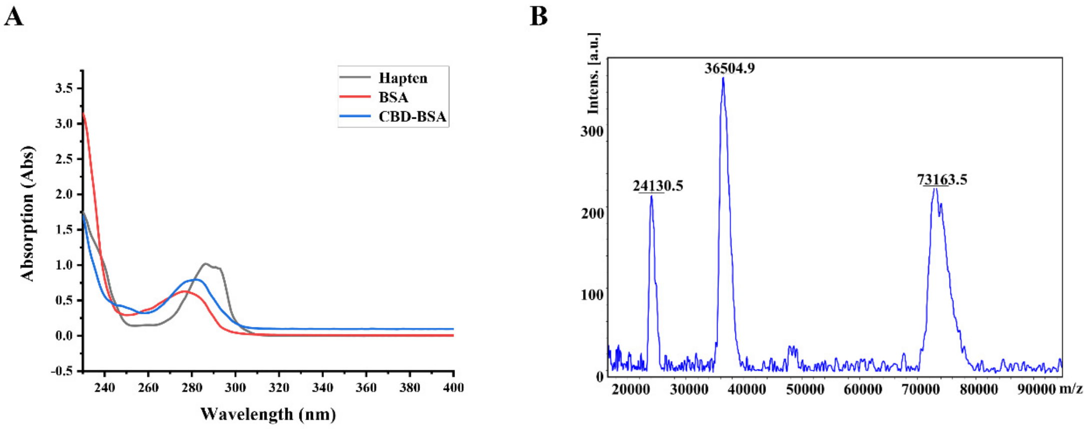

Figure S1. The characterization of immunogen CBD-BSA. (**A**) The UV-vis absorption spectra of CBD-BSA. (**B**) The matrix-assisted laser desorption time-of-flight (MALDI-TOF) of CBD-BSA.

Production of Monoclonal Antibody

Balb/c female mice were immunized with the CBD-BSA immunogen for mAb production. The mouse with the most sensitive antibody was selected for the cell fusion. Mouse no. 3 was sacrificed for fusion (Figure S2).

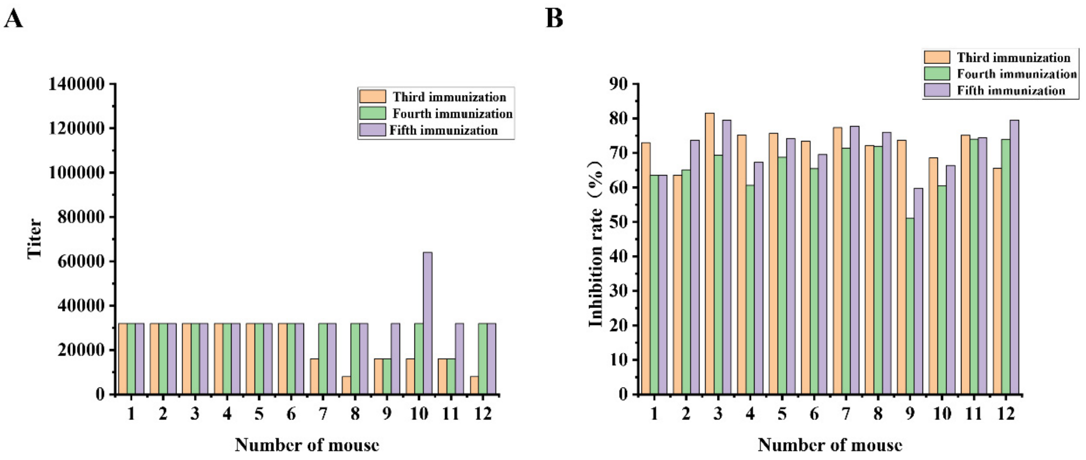

Figure S2. Effect of polyclonal antibody serum in immunized mouse. (**A**) Comparison of titers of polyclonal antibody serum in immunized mouse. (**B**) Comparison of CBD inhibition rates of polyclonal antibody serum in immunized mouse.

Reading of Test Strip Results

The results of the Au@PDAs-QDs-LFIA can be interpreted in the colorimetry mode or the fluorescence mode. The interpretation criteria in the colorimetry mode are as follows: as shown in Figure S3A, when there is no carbendazim in the liquid to be tested or the concentration is too low to be detected, red bands appear on C line and T line, the result is negative; When a detectable amount of carbendazim exists in the liquid to be tested, red band appeared on C line, weak red band appeared on T line, or no band appeared, the reading is positive. When no red band appears on C line, the result is invalid. The interpretation criteria in the fluorescence mode are as follows: as shown in Figure S3B, when the red band can be observed under natural light on C line, and there is no fluorescence on T line, the result is negative; When there is the red band on C line and fluorescence on T line, the result is positive. If no red band is observed under natural light of C line, the result is invalid.

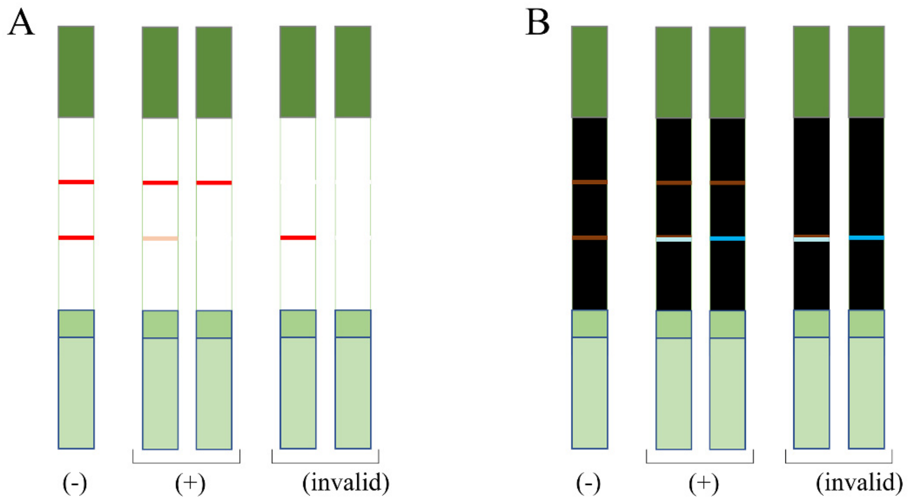

Figure S3. Schematic diagram of Au@PDAs-QDs-LFIA test strip for result judgment.

Characterization of ZnCdSe/ZnS QDs and QDs-OVA

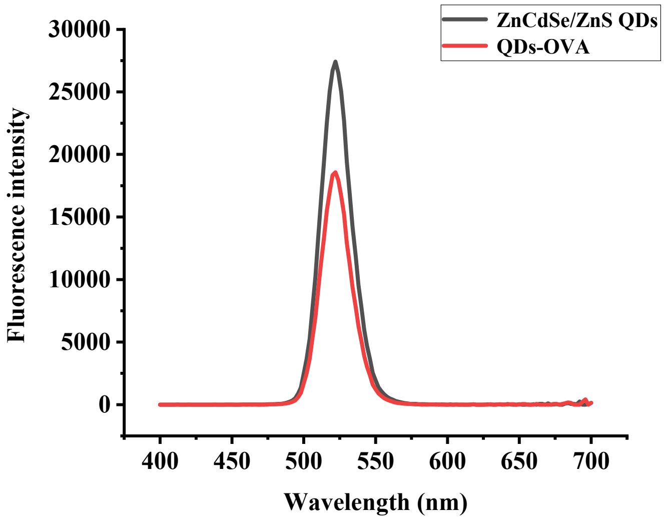

Figure S4. Photoluminescence spectra of ZnCdSe/ZnS QDs and QDs-OVA.

Characterization of Au@PDAs-mAb and QDs-OVA

The Au@PDAs-mAb and QDs-OVA solutions with different storage times were characterized by UV-vis absorption spectrum and photoluminescence spectrum. As shown in Figure S5, they both show good dispersion and Au@PDAs-mAb has the same absorption peak at 535 nm, while the QDs-OVA has a similar fluorescent intensity after a 30 days storage. These results indicated that the two probes had good colloidal stability and optical stability.

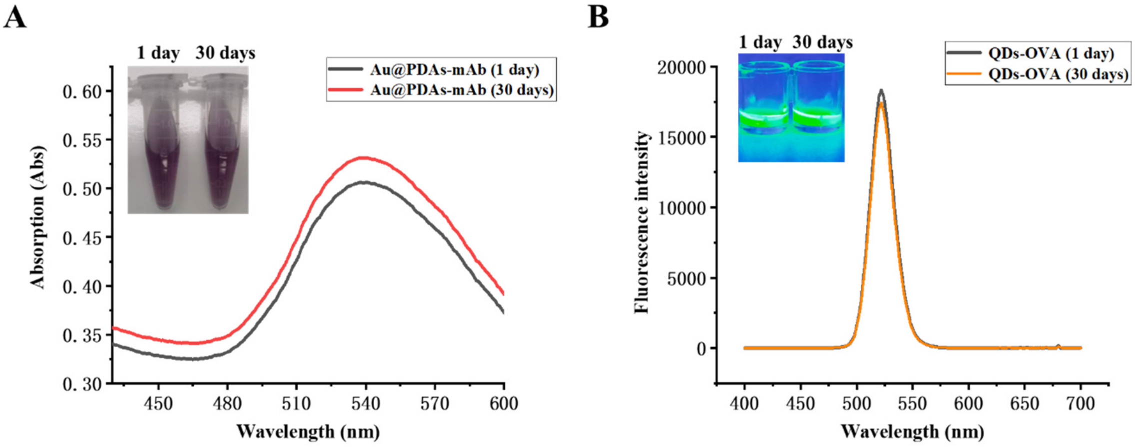

Figure S5. (**A**) UV-vis absorption spectrum of Au@PDAs-mAb. (**B**) Photoluminescence spectrum of QDs-OVA.

Optimal pH Value and Amount of mAb for Detection Probe

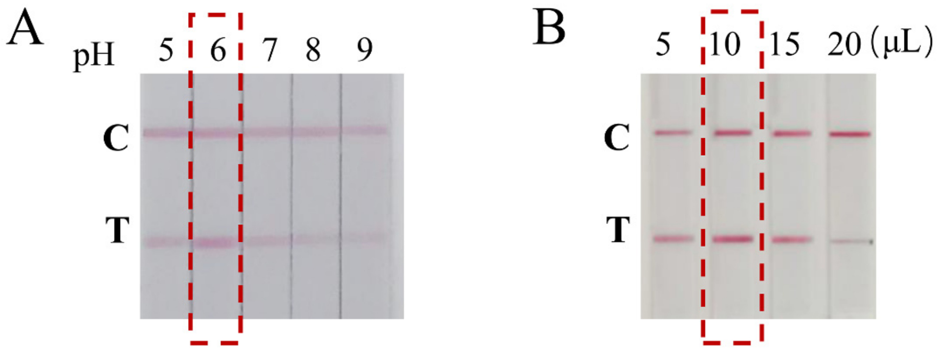

Figure S6. Optimal pH value and amount of mAb for detection probe. (**A**) Optimal pH value for anti-CBD mAb is 6. (**B**) Optimal amount of mAb for anti-CBD mAb is 10 μL (1.01 μg/mL).

Optimization of the Au@PDAs-QDs-LFIA Strip

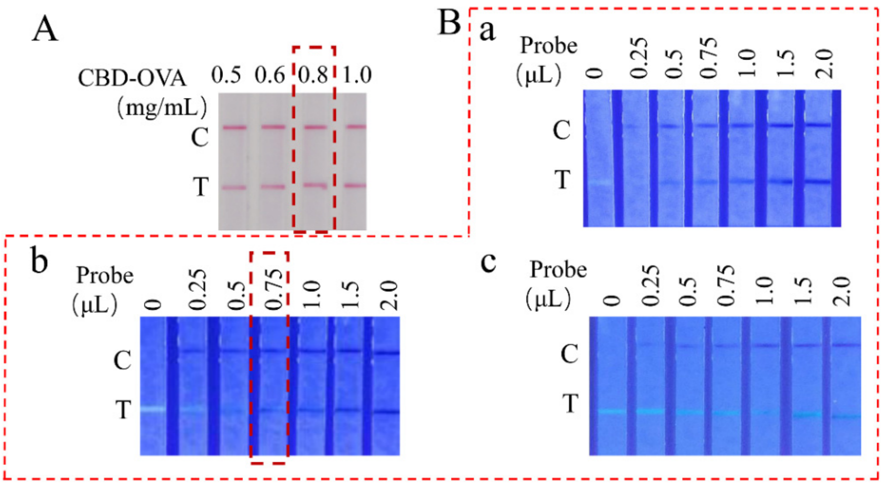

Figure S7. Optimization of test strip spraying conditions. (**A**) Detection results of strip with 0.5, 0.6, 0.8, 1.0 mg/mL CBD-OVA. (**B**) Fluorescence quenching of strip with 0.8 mg/mL CBD-OVA+200, 400, and 600 nM QDs-OVA after adding different amounts of probes.

Optimization of Test Conditions for Samples

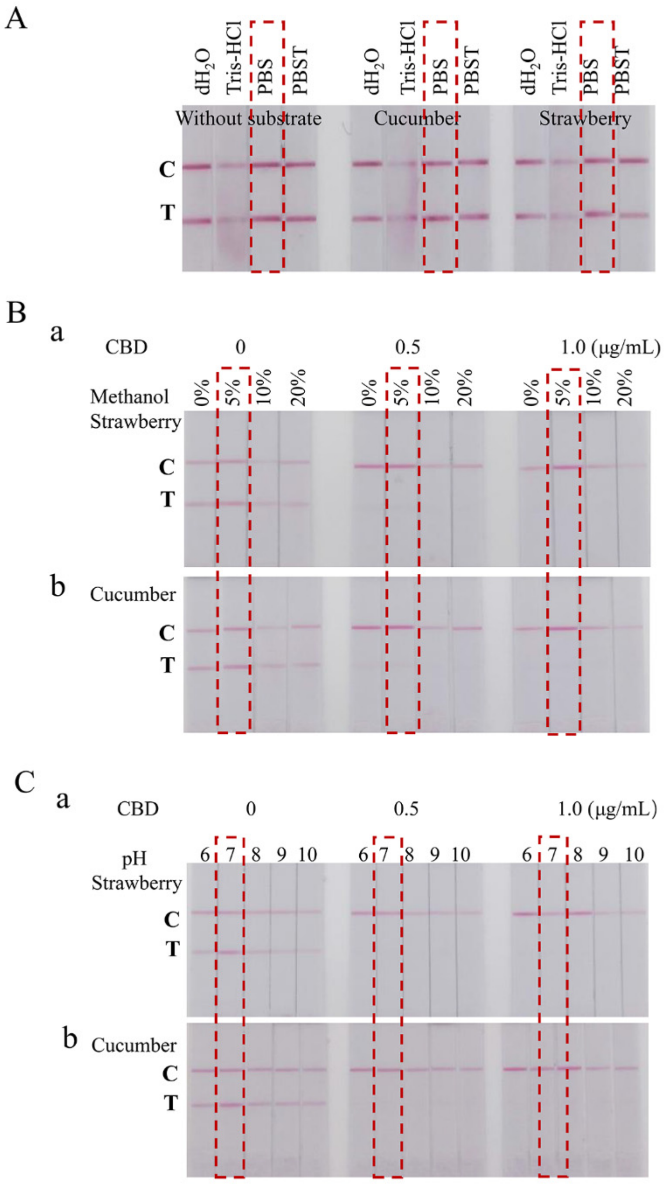

Figure S8. (**A**) Test strip chromatographic results of four sample diluents of dH_2O, Tris-HCl, PBS, and PBST. (**B**) Test results of strawberry (a) and cucumber (b) matrix samples diluted with diluents of different methanol content. (**C**) Test results of strawberry (a) and cucumber (b) matrix samples diluted with diluents of different pH.
